# Factors associated with needle sharing among people who inject drugs in Yunnan, China: a combined network and regression analysis

**DOI:** 10.1186/s40249-016-0169-y

**Published:** 2016-08-09

**Authors:** Xin Chen, Lin Zhu, Yan-Heng Zhou, Feng-Liang Liu, Hong Li, Zhi-Hong Yao, Lin Duo, Wei Pang, Mei Ye, Yong-Tang Zheng

**Affiliations:** 1 Key Laboratory of Animal Models and Human Disease Mechanisms of the Chinese Academy of Sciences and Yunnan Province, Kunming Institute of Zoology, Chinese Academy of Sciences, Kunming, Yunnan 650223 China; 2 Yunnan Center for Disease Control and Prevention, Kunming, Yunnan 650022 China; 3 The Second People’s Hospital of Yunnan Province, Kunming, Yunnan 650021 China; 4 Kunming College of Life Science, University of Chinese Academy of Sciences, Kunming, Yunnan 650204 China; 5 College of Life Sciences, Yan’an University, Yan’an, Shaanxi 716000 China

**Keywords:** People who inject drugs, Needle sharing, Risk behaviours, Network analysis, Regression analysis, China

## Abstract

**Background:**

Network analyses have been widely utilized to evaluate large datasets, but have not yet been used to explore factors associated with risk behaviours. In combination with traditional regression analysis, network analyses may provide useful information and highlight key factors for reducing needle sharing behaviours among people who inject drugs (PWID).

**Methods:**

Sociodemographic data, and information on injection behaviour and sexual practices were collected from a cross-sectional survey that was conducted with PWID in five prefectures of Yunnan province, China. A combination of logistic regression and correlation network analyses were used to explore key factors for reducing needle-sharing behaviours among PWID.

**Results:**

In a total of 1 049 PWID, 37.5 % had a history of needle or syringe sharing. The logistic analysis showed that Zhaotong, Qujing, Dehong, or Lincang residents, diazepam use, longer injection duration, needle reuse, and infection with HIV, viral hepatitis, tuberculosis and/or malaria were independently associated with needle sharing. The correlation network analyses showed that, compared to PWID who had never shared needles, PWID who did share needles would achieve harm reduction goals faster and more permanently. HIV serostatus and marital status were found to be closely associated with other risk factors. By combining regression analyses with network analyses, it was shown that PWID who are HIV seropositive will be an ideal target group for harm reduction programs.

**Conclusion:**

Needle-sharing behaviours are common among PWID in Yunnan, and harm reduction programs may help PWID who are HIV seropositive reduce risk behaviours and prevent blood borne diseases.

**Electronic supplementary material:**

The online version of this article (doi:10.1186/s40249-016-0169-y) contains supplementary material, which is available to authorized users.

## Multilingual abstract

Please see Additional file 1 for translations of the abstract into the five official working languages of the United Nations.

## Background

Bordering north-eastern Myanmar, northern Laos, and northern Vietnam, Yunnan is a region that plays a crucial role in the transmission of blood borne infectious diseases in the Lancang-Mekong subregion. According to recent studies, drugs produced in the ‘Golden Triangle’ region, which consists of eastern Myanmar, western Laos, and northern Thailand, are exported from Myanmar to Yunnan [1]. Along this drug route, subtypes B and C of the human immunodeficiency virus type 1 (HIV-1) are transmitted; subtypes B, C, and B/C recombinants are transmitted through the northwest of China, particularly through the Xinjiang province [1–4]. Studies have also shown that the circulating form of HIV, CRF01_AE, was introduced into Yunnan from northern Vietnam, likely through cross-border commercial sexual activities [5]. In 2012, a study showed that unique recombinant forms of HIV-1 were transmitted to Myanmar from Yunnan in the early 1990s [6]. These findings suggest that Yunnan may be a ‘transfer station’ of multidirectional transmission of HIV between China and its bordering countries.

People who inject drugs (PWID) are at a high risk of contracting blood borne diseases, and needle-sharing behaviours are believed to be the main route of transmission. The percentage of those who share needles among PWID ranges from 16 to 76 % in different countries, and is approximately 37 % in some countries [7–11]. Therefore, higher rates of needle or paraphernalia sharing can lead to an increased prevalence of blood borne diseases, including HIV, and hepatitis B and C virus infections [12, 13]. Although sharing of injecting equipment has been shown among PWID in Yunnan, there is a lack of up-to-date data in this field. As blood borne virus prevalence can be an indicator of needle and syringe sharing, a new evaluation of HIV prevalence in this population can be used as a measure of levels of needle and syringe sharing. Furthermore, reliable data on factors associated with needle and syringe sharing among PWID in Yunnan are scarce. Accurate information in this field is crucial for developing effective harm reduction strategies, reducing needle and syringe sharing among PWID, and lowering the disease burden of blood borne viruses.

Most previous studies have used traditional methods (including logistic regression) to investigate factors associated with needle and syringe sharing, and findings of these studies have contributed greatly to reducing needle-sharing behaviours among PWID. However, the interaction between each factor cannot be explored by traditional methods. Network analysis, which has been widely utilized in larger datasets in fields such as public health, social sciences, international relations, and protein dynamics, has contributed greatly to dealing with large datasets [14–17]. Network analyses can thoroughly and elegantly interpret large datasets into visual figures, show the inherent complexity of factor interactions, and explore the rate of influence that a given factor has in a correlation network. Therefore, using network analysis to analyse needle-sharing behaviours among PWID may provide useful information and highlight key factors for harm reduction programs.

In the present study, 1 049 PWID were recruited from Yunnan province in 2009; 20 sociodemographic and drug use behaviour factors were assessed. A combination of regression and network analyses was performed to evaluate needle-sharing characteristics, examine the possible predictors of needle sharing, compare the influence of each factor on PWID who partake and do not partake in needle-sharing behaviours, and explore key factors necessary for reducing needle-sharing behaviours.

## Methods

### Study participants and data collection

**Fig. 1:**
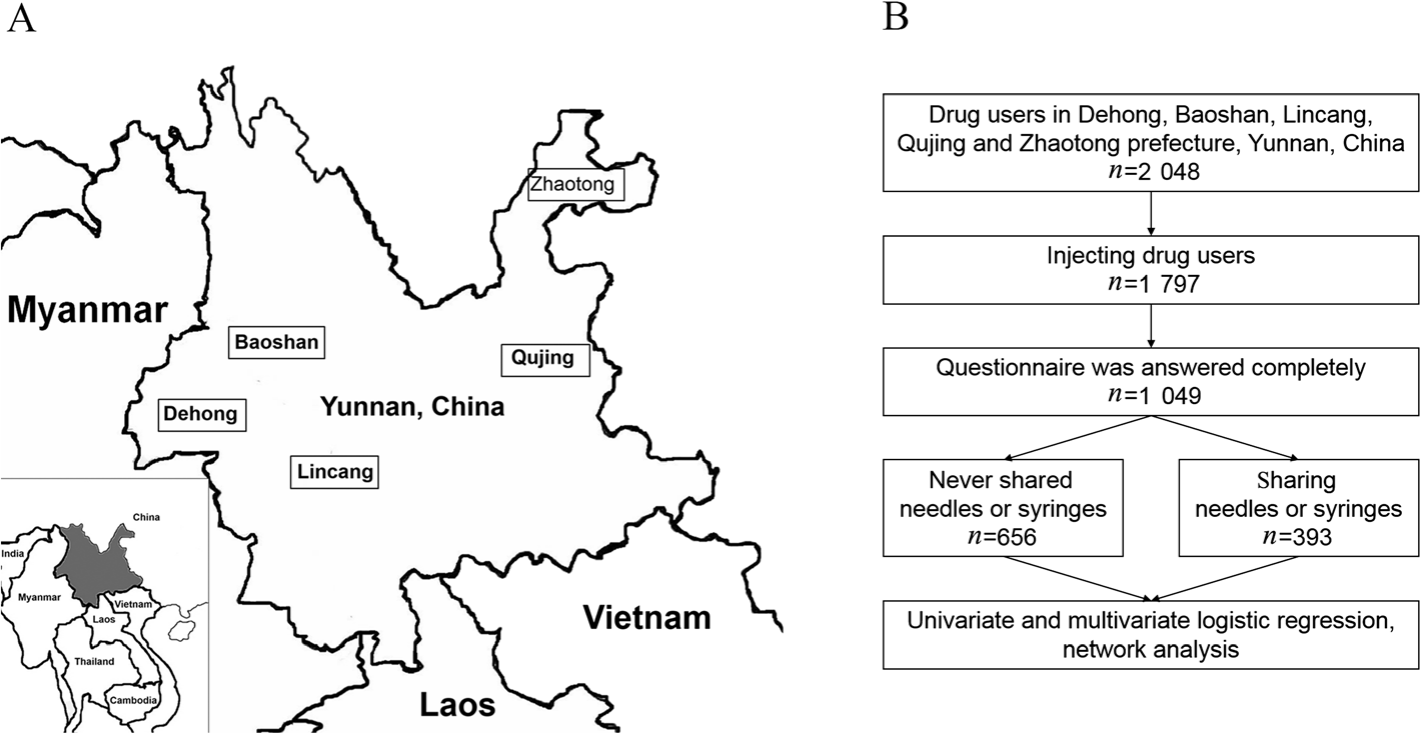
The sampling sites (**a**) and flow chart (**b**)

From March to December 2009, a cross-sectional survey was conducted with drug users from urban detoxification centres in the Dehong, Baoshan, Lincang, Qujing, and Zhaotong prefectures of Yunnan province, China (see Fig. 1). Once written informed consent was obtained, anonymous questionnaire-based interviews were conducted. The collected data included baseline sociodemographic characteristics, information on drug use and sexual behaviours, and whether the respondent was diagnosed with HIV and/or other infectious diseases (see Table 1). Only those who met the following criteria were selected as study subjects: 1) had a history of intravenous drug use, and 2) answered all the questions in the questionnaire (see Fig. 1).

**Table 1:** Basic characteristics of needle-sharing behaviors among PWID in Yunnan, China

	Total	Those who shared needles	Univariate logistic regression		Multivariate logistic regression	
	n(%)	n(%)	*OR* (95 % *CI*)	*P-*value^a^	*OR* (95 % *CI*)	*P - *value
Place of residence				7.7E^−25^		1.9E^−10^
Baoshan	340(32.4)	51(15.0)	Ref^b^		Ref	
Zhaotong	381(36.3)	157(41.2)	4.0(2.8–5.7)	6.9E^−14^	3.6(2.4–5.7)	3.8E^−09^
Qujing	112(10.7)	56(50.0)	5.7(3.5–9.1)	8.4E^−13^	5.9(3.4–10.3)	2.7E^−10^
Dehong	140(13.3)	74(52.9)	6.4(4.1–9.9)	4.3E^−16^	3.0(1.7–5.2)	1.2E^−04^
Lincang	76(7.2)	55(72.4)	14.8(8.3–26.6)	1.5E^−19^	4.9(2.3–10.1)	2.5E^−05^
Gender				5.4E^−01^		
Male	1 018(97.0)	383(37.6)	Ref			
Female	31(3.0)	10(32.3)	0.8(0.4–1.7)			
Age				3.5E^−01^		
13–25	200(19.1)	71(35.5)	Ref			
26–30	321(30.6)	116(36.1)	1.0(0.7–1.5)	8.8E^−01^		
31–35	284(27.1)	119(41.9)	1.3(0.9–1.9)	1.6E^−01^		
36–68	244(23.3)	87(35.7)	1.0(0.7–1.5)	9.7E^−01^		
Ethnicity				3.1E^−03^		
Han	852(81.2)	301(35.3)	Ref			
Minority^c^	197(18.8)	92(46.7)	1.6(1.2–2.2)			
Occupation				1.6E^−02^		
Various jobs^d^	226(21.5)	67(29.6)	Ref			
Farmer	362(34.5)	137(37.8)	1.4(1.0–2.1)	4.3E^−02^		
Jobless	461(43.9)	189(41.0)	1.6(1.2–2.3)	4.0E^−03^		
Marital status				2.3E^−01^		
Unmarried	485(46.2)	197(40.6)	Ref			
Married	387(36.9)	131(33.9)	0.7(0.6–1.0)	4.1E^−02^		
Cohabitated	62(5.9)	22(35.5)	0.8(0.5–1.4)	4.4E^−01^		
Divorced or widowed	115(11.0)	43(37.4)	0.9(0.6–1.3)	5.3E^−01^		
Education				8.9E^−04^		
None	67(6.4)	38(56.7)	Ref			
1–5 years	348(33.2)	143(41.1)	0.5(0.3–0.9)	1.9E^−02^		
6–9 years	488(46.5)	165(33.8)	0.4(0.2–0.7)	3.7E^−04^		
> 10 years	146(13.9)	47(32.2)	0.4(0.2–0.7)	8.3E^−04^		
Drug of choice				6.4E^−01^		
Heroin	1 010(96.3)	377(37.3)	Ref			
Heroin + others^e^	39(3.7)	16(41.0)	1.2(0.6–2.2)			
Other drug use				5.9E^−12^		2.1E^−04^
No	221(21.1)	46(20.8)	Ref		Ref	
Diazepam	450(42.9)	168(37.3)	2.3(1.6–3.3)	2.1E^−05^	1.6(1.0–2.6)	4.1E^−02^
Diazepam + others^f^	325(31.0)	167(51.4)	4.0(2.7–5.9)	3.0E^−12^	2.7(1.6–4.3)	1.0E^−04^
Other^f^	53(5.1)	12(22.6)	1.1(0.5–2.3)	7.7E^−01^	0.9(0.4–2.2)	8.0E^−01^
Method of drug intake				3.2E^−02^		
Injection	496(47.3)	169(34.1)	Ref			
Injection + oral	553(52.7)	224(40.5)	1.3(1.0–1.7)			
Duration of drug abuse				2.4E^−07^		
< 5 years	324(30.9)	85(26.2)	Ref			
6–10 years	281(26.8)	102(36.3)	1.6(1.1–2.3)	7.7E^−03^		
11–15 years	281(26.8)	124(44.1)	2.2(1.6–3.1)	4.7E^−06^		
16–20 years	163(15.5)	82(50.3)	2.8(1.9–4.2)	2.0E^−07^		
Drug use frequency (per day)				8.3E^−05^		
< 1	99(9.4)	17(17.2)	Ref			
2	334(31.8)	118(35.3)	2.6(1.5–4.7)	8.4E^−04^		
3	390(37.2)	164(42.1)	3.5(2.0–6.1)	1.1E^−05^		
> 4	226(21.5)	94(41.6)	3.4(1.9–6.2)	3.6E^−05^		
Most recent drug use				3.0E^−02^		
< 1 year	624(59.5)	225(36.1)	Ref			
1 year	336(32.0)	123(36.6)	1.0(0.8–1.3)	8.7E^−01^		
> 2 years	89(8.5)	45(50.6)	1.8(1.2–2.8)	9.0E^−03^		
Duration of drug use (injection)				7.7E^−13^		5.3E^−04^
< 5 years	499(47.6)	129(25.9)	Ref		Ref	
6–10 years	464(44.2)	216(46.6)	2.5(1.9–3.3)	3.5E^−11^	1.7(1.2–2.4)	1.2E^−03^
> 11 years	86(8.2)	48(55.8)	3.6(2.3–5.8)	8.2E^−08^	2.6(1.4–4.8)	2.0E^−03^
Source of syringe				8.8E^−06^		
Clinic	145(13.8)	26(17.9)	Ref			
Hospital	123(11.7)	51(41.5)	3.2(1.9–5.7)	3.3E^−05^		
Drugstore	692(66.0)	276(39.9)	3.0(1.9–4.8)	1.4E^−06^		
Other^g^	89(8.5)	40(44.9)	3.7(2.1–6.8)	1.4E^−05^		
Average reuse times per needle				1.6E^−23^		5.0E^−14^
1	638(60.8)	164(25.7)	Ref		Ref	
2	277(26.4)	134(48.4)	2.7(2.0–3.6)	3.6E^−11^	2.3(1.6–3.2)	3.3E^−06^
> 3	134(12.8)	95(70.9)	7.0(4.7–10.6)	2.0E^−20^	6.1(3.8–10.0)	2.1E^−13^
Number of sexual partners				2.1E^−01^		
None	121(11.5)	45(37.2)	Ref			
1	312(29.7)	103(33.0)	0.8(0.5–1.3)	4.1E^−01^		
2–3	285(27.2)	110(38.6)	1.1(0.7–1.6)	7.9E^−01^		
4–6	152(14.5)	56(36.8)	1.0(0.6–1.6)	9.5E^−01^		
7–10	59(5.6)	23(39.0)	1.1(0.6–2.0)	8.2E^−01^		
> 11	120(11.4)	56(46.7)	1.5(0.9–2.5)	1.4E^−01^		
STD diagnosis				7.6E^−07^		
No	867(82.7)	295(34.0)	Ref			
Yes	182(17.3)	98(53.8)	2.3(1.6–3.1)			
HIV serostatus				9.9E^−24^		1.3E^−06^
Negative	853(81.3)	254(29.8)	Ref		Ref	
Positive	196(18.7)	139(70.9)	5.8(4.1–8.1)		3.1(2.0–4.9)	
Diagnosis of other infectious disease^h^				3.3E^−12^		1.3E^−04^
No	718(68.4)	219(30.5)	Ref		Ref	
Yes	160(15.3)	97(60.6)	3.5(2.5–5.0)	4.1E^−12^	2.5(1.6–3.8)	2.6E^−05^
Unknown	171(16.3)	77(45.0)	1.9(1.3–2.6)	3.3E^−4^	1.3(0.9–2.0)	1.8E^−01^

^a^Using scientific notation

^b^Reference

^c^Achang, Bai, Zang, Dai, De'ang, Hui, Jingpo, Lisu, Man, Mian, Miao, Tujia, Yi, Zhuang

^d^Cook, electrician, barker, waiter, government staff, businessman, nurse, worker, chauffeur, builder, hotel manager, miner, carpenter, repairman, salesman, doctor

^e^Ketamine, methamphetamine, ecstasy, opium, ephedrine, marijuana

^f^Pethidine hydrochloride, triazolam, methadone

^g^Needle exchange programs, Centers for Disease Control and Prevention, store, friends

^h^Hepatitis A virus, hepatitis B virus, hepatitis C virus, tuberculosis, malaria

Five milliliters of venous blood was collected from each study subject using vacutainer tubes containing EDTA-2 K. Blood samples were centrifuged and serum was stored at −80 °C in the freezer until HIV antibody testing was performed. The HIV serostatus was assessed using a diagnostic kit for antibody to HIV (1 + 2) (Colloidal Gold) (Alere Medical Co., Ltd, Chiba, Japan), and those who tested positive were confirmed using a diagnostic kit for antibodies to HIV (1 + 2) (enzyme-linked immunosorbent assay) (Wantai Medical Co., Ltd, Beijing, China), according to the manufacturers’ instructions.

All the data obtained from the questionnaires and previous laboratory tests were used in the following analyses. To explore the key factors for reducing needle-sharing behaviors among PWID, participants were divided into two subgroups: those who had never shared needles were allocated to the never sharing needles group (NSNG) and those who had a history of needle sharing were allocated to the sharing needles group (SNG) (see Fig. 1).

### Regression analyses

To compare the differences between PWID in different groups (NSNG and SNG), Student’s t-tests (two-tailed) were used for the factor of age and Mann–Whitney U tests (two-tailed) were used for the factors of gender, ethnicity, occupation, marital status, and education level.

A univariate logistic regression analysis was conducted to compare the percentage of those PWID who shared needles between the NSNG and the SNG. Variates with *P*-values less than 0.05 were included in the multivariate logistic regression model to explore independent predictors of needle sharing.

All analyses mentioned above were performed using the statistical software Statistical Package for Social Sciences (SPSS, version 22.0, IBM corporation, Armonk, USA) and a *P*-value of <0.05 was considered statistically significant.

### Network analyses

The correlation coefficient between each factor was calculated using SPSS v22.0, and network analyses of the NSNG and the SNG were performed using Cytoscapev 2.8.3 (http://www.cytoscape.org/) [18, 19]. Based on correlation coefficients above 0.1 or below −0.1, and *P*-values less than 0.05, a network of correlation profiles consisting of the connections between each risk factor were generated, with nodes corresponding to risk factors and edges to pairs of risk factors that were significantly correlated. Network statistics that were used to describe global and local network properties were calculated and edges were treated as undirected as they represent mutual interactions. The calculation of global network properties contained the number of nodes and edges, the average number of neighbours, the network diameter, density, centralization, heterogeneity, clustering coefficient, and the characteristic path length. The calculation of local network properties contained the betweenness centralities (BCs) and closeness centralities (CCs) of each node. Random network statistics were also calculated from 1 000 randomly generated networks that had the same number of nodes and edges as the empirical networks, and were randomly assembled using the Erdös–Rényi model networks. Network module identification was performed using the MCODE v1.3 plugin for Cytoscape [20].

## Results

### Sociodemographic characteristics of study participants

Initially, 2 048 drug users were recruited from the Dehong, Baoshan, Lincang, Qujing, and Zhaotong prefectures in Yunnan. Among them, 1 797 were PWID, but only 1 049 successfully completed the questionnaire (see Fig. 1). Most of the participants were male (97.0 %), of the Han ethnicity (81.2 %), aged between 20 and 40 years (88.1 %), and had school experience (93.6 %). Out of all the participants, 18.7 % were HIV seropositive and 15.3 % self-reported to have other infectious diseases, including hepatitis A, B, or C; tuberculosis; and/or malaria (see Table 1).

Among the 1 049 PWID, 37.5 % (*n* = 393) had a history of needle sharing. Among these, 13.2 % (*n* = 52) shared needles with those known to be HIV seropositive. No differences were observed between the NSNG and the SNG in terms of age (30.77 ± 7.17 versus 30.95 ± 6.93; *P* =0.349), gender (*P* = 0.543), or marital status (*P*=0.230). Compared to PWID with a needle-sharing history, those without a needle-sharing history were more likely to be of the Han ethnicity, have full-time jobs, and be highly educated (*P* < 0.01).

### Factors associated with needle sharing

Because such a high percentage of needle sharing among PWID was observed, a univariate logistic regression analysis was conducted to compare the different percentage of PWID who partake in needle-sharing behaviours between different substratification of each factor (see Table 1). Results showed that needle sharing was not significantly correlated with age, gender, or marital status of a drug user, however, the PWID who were jobless, belonged to a minor ethnicity, had a low education, and lived in Myanmar-bordering prefectures (Dehong and Lincang) had a greater tendency toward needle-sharing behaviours. A longer duration and greater frequency of drug abuse/injection increased the possibility of sharing needles. Moreover, PWID who combined injecting drugs with oral intake, bought needles from hospital, drugstore or other resources, or reused needles were found to share needles more often. In addition, use of diazepam and heroin facilitated needle sharing, while using other drugs did not. Lastly, PWID who had been diagnosed with sexually transmitted diseases (STDs), HIV, or other infectious diseases (hepatitis, tuberculosis, and/or malaria) were more likely to share needles, suggesting that sharing needles might transmit blood borne diseases.

All 15 factors related to needle sharing were included in the multivariate logistic regression analysis (see Table 1). Of these, living in Zhaotong, Qujing, Dehong or Lincang, using diazepam, injecting for a long time, reusing needles often, and being HIV positive and having other infectious diseases, such as hepatitis, tuberculosis, and/or malaria, were found to be independently associated with needle sharing. These results showed that the factors affecting needle-sharing behaviours were mainly due to drug use behaviours rather than sociodemographic factors, verifying that contracting blood borne diseases (e.g. HIV, viral hepatitis) might be the main consequence of needle sharing.

### The global and local properties of correlation networks of PWID in the NSNG and the SNG

**Fig. 2:**
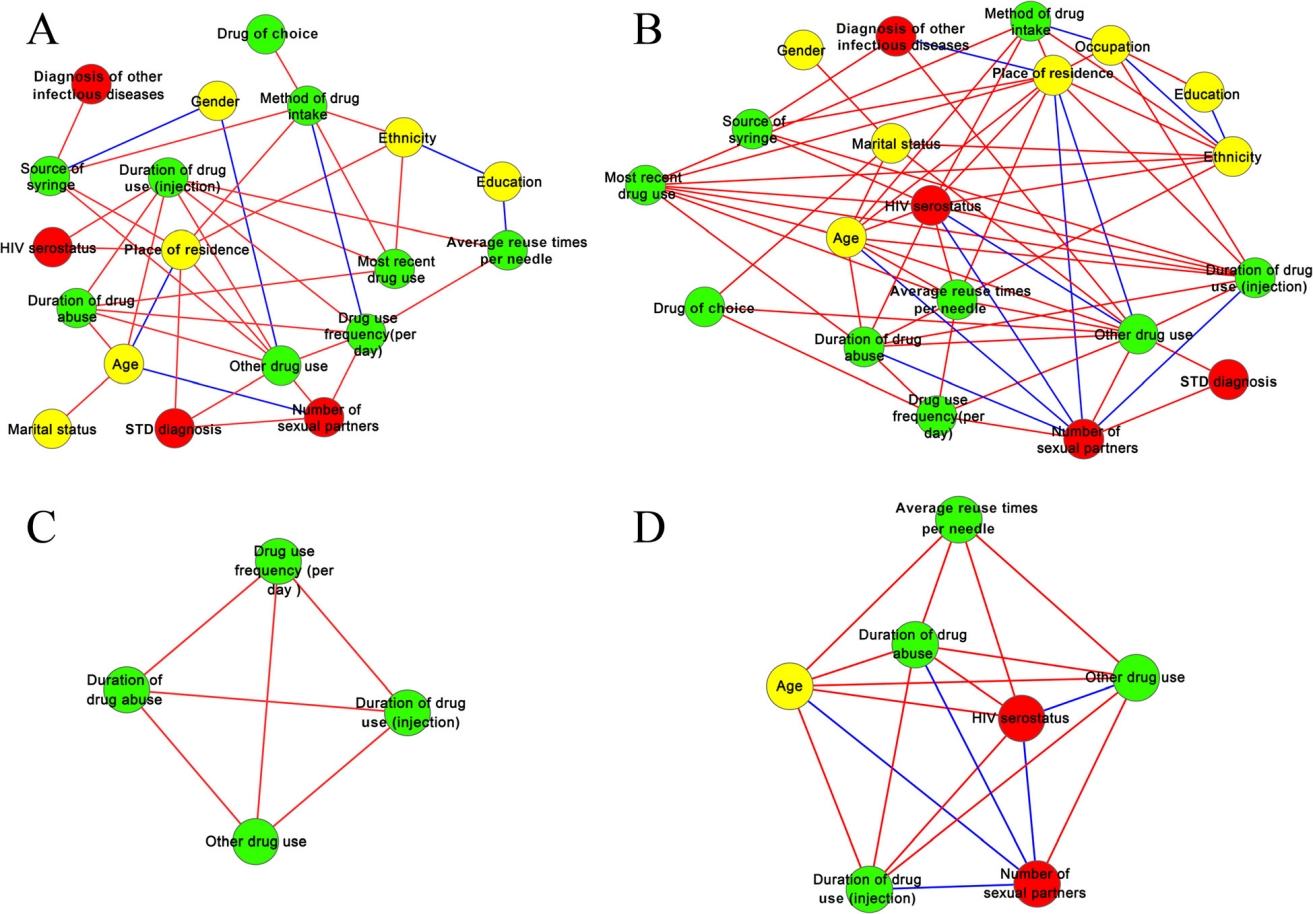
The correlation network and network modules of PWID who did not have (**a**, **c**) and who had (**b**, **d**) a history of needle sharing. Yellow, green, and red codes correspond to sociodemographic, drug use, and sexual/infectious disease factors, respectively. Red and blue edges correspond to positive and negative relations between each factor

Correlation networks that integrated sociodemographic, drug use, and sexual/infectious disease factors among NSNG and SNG were constructed (see Fig. 2). To determine whether the network statistics observed in the empirical correlation networks of both the NSNG and the SNG deviated from random connectivity and not observed by chance, the clustering coefficient and characteristic path length of the empirical correlation networks were compared to those of the Erdös–Rényi model of random networks (see Table 2). Results showed that both were significantly larger than those of the random networks (*P* < 0.01), indicating that the empirical correlation networks were all structured to non-random features.

**Table 2:** Global properties of empirical and random networks of the NSNG and the SNG

Global properties	Never shared needles	Sharing needles
Network clustering coefficient	0.273	0.588
Network clustering coefficient_Random_	0.221(±0.002)	0.364(±0.002)
Characteristic path length	2.140	1.858
Characteristic path length_Random_	2.125(±0.003)	1.726(±0.001)
Number of nodes	19.000	20.000
Number of edges	38.000	65.000
Average number of neighbours	4.000	6.500
Network density	0.222	0.342
Network diameter	4.000	4.000
Network heterogeneity	0.532	0.509
Network centralization	0.248	0.322
Network clustering coefficient	0.273	0.588
Characteristic path length	2.140	1.858

In the correlation network of the SNG, the number of sexual partners was negatively correlated with residence, age, duration of drug abuse/injection, and HIV serostatus, indicating that long-term, frequent drug use might inhibit sexual desire, and that the predominant route of HIV transmission among PWID could be needle sharing (through blood).

The module in the correlation network of the NSNG and the SNG consisted of four and seven hub nodes, respectively, which were involved in many interactions and represented functionally important factors. Changes in hub nodes could affect the most nodes in their vicinity, as well as the whole structure of the correlation network.

In comparison to the NSNG, the network size, average number of neighbours, network density, and network clustering coefficient of the SNG were larger, while the network heterogeneity was lower (see Table 2), indicating that the correlation among each risk factor in the SNG was higher. In addition, as the diameter of the correlation network in both groups was equal, the characteristic path length of the correlation network of the SNG was lower. Changes in each risk factor could permeate the whole correlation network more quickly in the SNG by minimizing the transition time between the interactions of each factor. Paired with higher network centralization, a lower characteristic path length implied that the resilient ability of changes in the network of the SNG was also lower. Overall, these results suggest that the global properties of correlation networks vary between the NSNG and the SNG, and that harm reduction measures aimed at the SNG could achieve goals in a more rapid and durable manner.

Comparative analyses of the correlation networks revealed substantial differences in global network properties, but could not provide a comprehensive understanding of the impact of a given node in the correlation network. Therefore, BCs and CCs were calculated to quantify the topological importance of a given node (see Table 3). Residence, duration of drug injection, other drug use, age, and ethnicity were above average for BCs and CCs for all nodes in the NSNG and the SNG, while method of drug intake, drug use frequency per day, and source of syringe were only above average for BCs and CCs in the NSNG, and HIV serostatus and marital status were only above average for BCs and CCs in the SNG. Among them, the factors of residence and other drug use had the highest BCs and CCs in the NSNG and the SNG, respectively, indicating that these factors had a stronger influence over the correlation networks than the other nodes, and that changes in their characteristics could permeate the correlation network more quickly. These results were also found intuitively in the correlation networks.

**Table 3:** BCs and CCs in the NSNG and the SNG

	Never shared needles		Sharing needles	
	BC value (rank)	CC value (rank)	BC value (rank)	CC value (rank)
Residence	0.221(1)	0.600(1)	0.127(3)	0.704(1)
Other drug use^a^	0.176(2)	0.581(2)	0.209(1)	0.704(1)
Method of drug intake	0.174(3)	0.581(2)	0.007(14)	0.528(12)
Source of syringe	0.146(4)	0.529(6)	0.004(17)	0.463(17)
Age	0.130(5)	0.514(8)	0.066(5)	0.655(4)
Duration of drug injection	0.125(6)	0.581(2)	0.056(7)	0.633(5)
Drug use frequency (per day)	0.097(7)	0.563(5)	0.011(13)	0.500(15)
Ethnicity	0.070(8)	0.500(9)	0.121(4)	0.613(6)
Times reusing needle	0.041(9)	0.439(14)	0.037(9)	0.613(6)
Duration of drug abuse	0.029(10)	0.529(6)	0.017(11)	0.594(8)
Most recent drug use	0.028(11)	0.486(10)	0.013(12)	0.576(10)
Number of sexual partners	0.022(12)	0.474(11)	0.045(8)	0.594(8)
Education	0.008(13)	0.375(16)	0(18)	0.404(19)
Ever diagnosed with a STD	0.005(14)	0.462(12)	0(18)	0.432(18)
HIV serostatus	0.003(15)	0.450(13)	0.066(5)	0.679(3)
Marital status	0(16)	0.346(19)	0.131(2)	0.559(11)
Occupation	-(-)^b^	- (-)^b^	0.031(10)	0.514(13)
Other infectious diseases^c^	0(16)	0.353(18)	0.006(15)	0.514(13)
Drug of choice	0(16)	0.375(16)	0.006(15)	0.475(16)
Gender	0(16)	0.409(14)	0(18)	0.365(20)

^a^Diazepam, pethidine hydrochloride, triazolam, or methadone

^b^Occupation not included in the correlation network of the NSNG

^c^Hepatitis A virus, hepatitis B virus, hepatitis C virus, tuberculosis, or malaria

### Key factors for reducing needle-sharing behaviours

Based on the regression analysis, six factors were found to be independently associated with needle sharing. From the network analysis, seven factors were above average for BCs and CCs for all nodes in the SNG and eight in the NSNG. All factors, a total of 12, could be targets for harm reduction measures. As these 12 factor-associated PWID would almost covered the entire population of PWID, it’s difficult to taking measures among this group.

To narrow down the key factors for reducing needle-sharing behaviours among PWID, combined network and regression analyses were conducted. It was elucidated that taking measures aimed at the SNG would achieve goals quicker and more successfully. The BCs and CCs of HIV serostatus and marital status were above average only in the SNG;HIV seropositivity was found to be associated with needle sharing behaviours, while any subcategory of marital status (unmarried, married, cohabitated, divorced, or widowed) was not.

In summary, HIV seropositivity is a unique factor that is associated with needle sharing and has a strong effect only on PWID who partake in needle-sharing behaviours. Therefore, those who are HIV seropositive would be the ideal target group for harm reduction programs.

## Discussion

Research on needle-sharing behaviours has been conducted across different provinces of China, and consequent findings have contributed tremendously to the development of successful harm-reduction programs. From January to April 2001, J. T. Lau et al. [21] administered a survey of 262 drug users in Shenzhen, Guangdong province, and found that 60.6 % of males and 45.3 % of females among PWID shared needles with others. From August 2003 to June 2004, Dick Chamla et al. [22] conducted a survey of 266 PWID in Chengdu, Sichuan, and found that 38.72 % had shared needles with their partners. In the present study, we systematically reported the characteristics of needle-sharing behaviours among PWID in the Yunnan province. Although the total prevalence of needle sharing in Yunnan was not significantly different compared to other provinces in China, the percentage of those who shared needles greatly varied across the different prefectures (15.0–72.4 %). We also found that a small quantity of PWID shared needles with partners who are known to be HIV seropositive. These results provide important information on the educational interventions that are immediately needed, and highlight which regions require the most attention.

Factors associated with needle sharing have been described in previous studies. For example, in 2007, Choi et al. reported that sex, ethnicity, having a primary partner who injects drugs, family support, and social discrimination were all significantly associated with needle sharing, based on a cross-sectional survey of 200 PWID in Sichuan, China [23]. Based on our results, six factors were found to be independently associated with needle sharing. The results did not significantly differ from those of previous studies, though larger numbers of participants and factors were taken into consideration.

By comparing the correlation networks of PWID in the NSNG and the SNG, we found that programs that attempt to reform the behaviours of PWID who partake in needle-sharing behaviours might be more successful than for PWID who do not partake in needle-sharing behaviours. Further, by calculating the BCs and CCs for each node of the NSNG and the SNG, we found that HIV serostatus and marital status have a strong effect on PWID in the SNG, making it possible to distinguish PWID who partake in needle-sharing behaviours from those who do not. Finally, by combining network analysis with regression analysis, HIV seropositivity was found to be a unique factor that had a strong effect only on PWID in the SNG, and was significantly associated with needle sharing. All these findings drew a clearer picture for harm reduction programs: taking actions aimed at PWID who are HIV seropositive may work better to reduce needle-sharing behaviours across all groups of PWID.

According to a study by M. Vazirian et al. [24], shared use of needles or syringes was significantly lower among PWID who received an estimated ≥7 syringes per week than those who did not [2.9 % versus 35.1 %; adjusted odds ratio (*OR*) = 14.36, 95 % confidence interval (*CI*) 2.30–89.56]. In China, the prevalence of HIV among PWID was approximately 10.9 % by 2011 (compared to 18.7 % in the present study), meaning that the HIV seropositive group was a relatively smaller group [25]. Therefore, focusing on PWID who are HIV seropositive can ensure that this group receives clean needles, especially in locations that cannot afford to distribute needles to all PWID. Free antiviral drugs are available in healthcare centres in China and HIV seropositive patients can receive them voluntarily, making it more convenient to provide PWID who are HIV seropositive with clean needles.

This study had several limitations. First, a timeframe was not applied to address PWID who most recently partook in drug injection and needle-sharing behaviours, influencing the timeliness of this study. Second, characteristics of harm reduction were not included in the questionnaire. Third, using hepatitis C virus as a predictor of a blood borne disease may have been better than using HIV, as the seroprevalence of hepatitis C is much higher than HIV among PWID. Despite these limitations, this study had evaluated the needle-sharing characteristics among PWID in Yunnan, and could provide useful information for the following harm reduction programs.

## Conclusions

In summary, based on a cross-sectional survey, we found that the percentage of those who shared needles among PWID in Yunnan was as high as reported in previous studies in other provinces in China. By combining regression and network analyses, PWID who are HIV seropositive were found to be the ideal target group for taking measures to reduce needle-sharing behaviours among PWID.

## Abbreviations

BC, betweenness centrality; CC, closeness centrality; HIV-1, human immunodeficiency virus type 1; NSNG, never sharing needles group; PWID, people who inject drugs; SNG, sharing needles group; STD, sexually transmitted disease.

## Additional file

Additional file 1: Multilingual abstracts in the five official working languages of the United Nations. (PDF 458 kb)
