# Supplementary material for: Factors associated with needle sharing among people who inject drugs in Yunnan, China: a combined network and regression analysis
Source: Infect Dis Poverty. 2016 Aug 9;5:73. doi: 10.1186/s40249-016-0169-y (PMC4977866; doi:10.1186/s40249-016-0169-y)
Supplement: Additional file 1: — Multilingual abstracts in the five official working languages of the United Nations. (PDF 458 kb) [file 40249_2016_169_MOESM1_ESM.pdf]

Translation of the abstract into the five official working languages of the United Nations

العوامل المرتبطة بمشاركة الإبر بين مدمني المخدرات عن طريق الحقن في يوننان، الصين: تحليل انحداري – شبكي مشترك.

خلفية: تم استخدام تحليل الشبكة على نطاق واسع لتقييم مجموعات كبيرة من البيانات، و لكن لم يسبق استخدامه لاستكشاف العوامل المرتبطة بالسلوكيات المحفوفة بالمخاطر. ان استخدام تحليل الشبكاة جنباً غلى جنب مع تحليل الإنحدار التقليدي من الممكن ان يوفر معلومات مفيدة و أن يسلط الضوء على العوامل الرئيسية للحد من السلوكيات المرتبطة بمشاركة الإبر بين متعاطي المخدرات عن طريق الحقن (PWID)

الطرق المتبعة: تم جمع بيانات اجتماعية – ديموغرافية و جمع معلومات حول سلوكيات الحقن و الممارسات الجنسية من دراسة مستعرضة أجريت على متعاطي المخدرات عن طريق الحقن (PWID) في خمس محافظات من مقاطعة يوننان- الصين.

تم استخدام تركيبة من تحليل الانحدار اللوجستي و تحليل الشبكة الارتباطي لاستكشاف العوامل الرئيسية المؤثرة في الحد من سلوكيات مشاركة الإبر بين متعاطي المخدرات

النتائج: في دراسة أجريت على إجمالي 1049 شخص من متعاطي المخدرات عن طريق الحقن أظهرت أن نسبة 37.5% كانت لديهم تجربة سابقة في مشاركة الإبر او المحاقن. التحليل اللوجستي أظهر بعض النتائج حول سكان مدن تشاوتونغ، تشوجينغ، ديهونغ و لينشانغ بما يفيد أن استخدام الديازيبام، الحقن لمدة أطول، إعادة استخدام الإبر و العدوى بمرض نقص المناعة البشرية HIV و التهاب الكبد الفيروسي و السل و الملاريا جميعها مرتبطة بمشاركة الإبر في تعاطي المخدرات.

أظهر تحليل الشبكة الإرتباطي أن هؤلاء الذين يشاركون الإبر قد يحرزون تقدماً أكبر و طويل الأمد في تحقيق اهداف الحد من الأضرار مقارنةً بأولئك الذين لم يشاركوا الإبر مسبقاً. وجد ايضاً أن الاختبارات المصلية لفايروس نقص المناعة المكتسب HIV و الحالة الاجتماعية مرتبطان بشكل وثيق بعوامل الخطر. بالجمع بين تحليل الإنحدار و التحليل الشبكي فقد تبين أن الأشخاص المصابون بفيروس نقص المناعة هم المجموعة المستهدفة الأولى في برامج الحد من الأضرار.

خلاصة: سلوكيات مشاركة إبر الحقن شائعة بين متعاطي المخدرات في مقاطعة يوننان، و من الممكن لبرامج الحد من الأضرار أن تساعد الأشخاص المصابين بفايروس نقص المناعة HIV على تعلم الحد من السلوكيات الخطيرة و منع الأمراض المنقولة عن طريق الدم.

Translated from English version into Arabic by Rola Majbor, through

TRANSLATORS
WITHOUT BORDERS

联合网络分析和回归分析探讨中国云南省静脉注射吸毒人群中共与用针具相关的因素

陈鑫，朱林，周衍衡，刘丰亮，李洪，姚志红，朵林，庞伟，叶梅，郑永唐

摘要

引用：网络分析已经广泛应用于大数据的处理，但尚未应用于分析与高危行为相关的因素。如果与传统的回归分析相结合，网络分析可能为减少静脉吸毒人群的共用针具行为提供更多有用的信息，并挑选出与之相关的关键因素。

方法：从对中国云南省 5 个州/市的静脉注射吸毒人群的横断面调查中，收集了人口统计学、注射吸毒以及性行为相关的数据。运用回归分析和网络分析相结合的方法，探讨与减少静脉注射吸毒人群共用针具行为相关的关键因素。

结果：在 1049 名静脉注射吸毒人员中，37.5%有过共用针具的行为。回归分析结果显示：住在昭通、曲靖、德宏或临沧，使用安定，注射吸毒时间更长，重复使用注射器，感染人类免疫缺陷病毒、病毒性肝炎、结核和/或疟疾与共用针具行为独立相关。网络分析结果显示：与不共用针具的注射吸毒人员相比，减少危害项目实施在共用针具的注射吸毒人群中将可以更快起作用，效果也能持续更长的时间；而且，人类免疫缺

陷病毒血清学特征和婚姻状态在共用针具的注射吸毒人群网络中与其它因素密切相关。联合回归分析和网络分析可以看出，感染人类免疫缺陷病毒的静脉注射吸毒人群是减少危害项目的理想靶向人群。
结论：共用针具行为在云南省静脉注射吸毒人群中十分普通。减少危害项目可以帮助感染人类免疫缺陷病毒的注射吸毒人员减少高危行为，降低感染血源性疾病的机率。

Translated from English version into Chinese by Chen Xin

Facteurs associés au partage d'aiguilles parmi les toxicomanes par injection au Yunnan (Chine) : analyse en réseau et de régression combinée

Xin Chen, Lin Zhu, Yan-Heng Zhou, Feng-Liang Liu, Hong Li, Zhi-Hong Yao, Lin Duo, Wei Pang, Mei Ye, and Yong-Tang Zheng

Résumé
Contexte : Les analyses en réseau sont largement utilisées pour évaluer les grands ensembles de donnés mais n'ont pas encore été employées pour explorer les facteurs associés aux comportements à risque. Associées à des analyses de régression classiques, elles peuvent apporter des informations utiles et mettre en lumière les facteurs essentiels permettant de réduire les partages d'aiguilles entre les consommateurs de drogues par injection.
Méthodes : Des données sociodémographiques et des informations sur les habitudes d'injection et les pratiques sexuelles ont été recueillies dans le cadre d'une étude transversale menée auprès de toxicomanes par injection dans cinq préfectures de la province chinoise du Yunnan. Une combinaison de régression logistique et d'analyse en réseau de corrélations a été utilisée pour explorer les principaux facteurs permettant de réduire le partage d'aiguilles entre ces consommateurs de drogues.
Résultats : Sur un total de 1049 toxicomanes par injection, 37,5 % avaient déjà partagé des aiguilles ou des seringues. L'analyse logistique a mis en évidence une association indépendante entre le partage d'aiguilles et les résidents de Zhaotong, Qujing, Dehong ou Lincang, la prise de diazépam, une durée de consommation prolongée, la réutilisation des aiguilles et l'infection par le VIH, les hépatites virales et/ou le paludisme. Les analyses en réseau de corrélation montrent que par rapport aux consommateurs qui n'ont jamais partagé d'aiguilles, ceux qui partagent leurs aiguilles atteindraient les objectifs de réduction des effets nocifs pour la santé plus rapidement et de façon plus permanente. Le statut sérologique du VIH et la situation maritale se sont avérés étroitement associés à d'autres facteurs de risque. En combinant des analyses de régression et des analyses en réseau, nous avons pu montrer que les toxicomanes par injection séropositifs pour le VIH constituent un groupe cible idéal pour les programmes de réduction des effets nocifs pour la santé.
Conclusion : Le partage d'aiguilles est fréquent parmi les toxicomanes par injection au Yunnan et des programmes de réduction des effets nocifs pour la santé pourraient aider ceux d'entre eux qui sont séropositifs pour le VIH à réduire les comportements à risque et à éviter la transmission de maladies par le sang.

Translated from English version into French by Suzanne Assenat, through

TRANSLATORS
WITHOUT BORDERS

Факторы, связанные с совместным использованием иглы потребителями инъекционных наркотиков в Юньнане, Китай: комбинированный сетевой/регрессивный анализ

Xin Chen, Lin Zhu, Yan-Heng Zhou, Feng-Liang Liu, Hong Li, Zhi-Hong Yao, Lin Duo, Wei Pang, Mei Ye, and Yong-Tang Zheng

Конспект

Обоснование: сетевой анализ широко использовался для оценки объемных пакетов данных, но не использовался для исследования факторов поведения, связанного с повышенным риском. В сочетании с традиционным регрессивным анализом сетевой анализ может дать полезную информацию и выделить ключевые факторы для уменьшения количества случаев совместного использования иглы потребителями инъекционных наркотиков (ПИН).

Методы: Путем перекрестного исследования собирались социально-демографические данные и информация о поведении и сексуальных наклонностях потребителей инъекционных наркотиков. Исследование проводилось среди ПИН в пяти префектурах китайской провинции Юньнан. Для выяснения ключевых факторов уменьшения привычки использования общего шприца среди ПИН использовались данные анализа сочетания логистического регресса и сетей.

Результаты: Всего было привлечено 1049 ПИН, 37,5% из которых имеют опыт использования общей иглы или шприца. Логистический анализ показал, что жители Чжаотуня, Цюйцзиня, Дегоня и Линьцаня (Zhaotong, Qujing, Dehong, и Lincang), которые используют диазепам, более продолжительные инъекции, пользуются одной иглой многократно, инфицированы ВИЧ, болеют вирусным гепатитом, туберкулезом и малярией, были независимо ассоциированы с совместным пользованием иглой. Корреляционный сетевой анализ показал, что по сравнению с ПИН, которые никогда не делили с кем-то одну иглу, те, кто пользуются иглой совместно, достигают целей по снижению вреда быстрее и более устойчиво. Положительный ВИЧ серологический статус и статус семейного положения, согласно исследованиям, оказались в тесной зависимости от других факторов риска. Комбинируя регрессивный анализ и сетевой анализ, можно увидеть, что ПИН с положительным серологическим статусом ВИЧ являются идеальной целевой группой для программ снижения вреда.

Вывод: Совместное использование иглы широко распространено среди ПИН Юньнаня. Программы снижения вреда могут быть полезны для ПИН с положительным серологическим статусом ВИЧ. Результатом применения программ может быть снижение риска и предотвращение распространения инфекций, передающихся через кровь.

Translated from English version into Russian by Anna Philippova, through

TRANSLATORS WITHOUT BORDERS

Factores asociados al hábito de compartir agujas en personas que se inyectan droga en Yunnan, China: un análisis combinado de regresión y redes

Xin Chen, Lin Zhu, Yan-Heng Zhou, Feng-Liang Liu, Hong Li, Zhi-Hong Yao, Lin Duo, Wei Pang, Mei Ye, y Yong-Tang Zheng

Resumen

Antecedentes: Los análisis de redes se han utilizado ampliamente para evaluar grandes conjuntos de datos, pero todavía no han sido utilizados para explorar los factores asociados a comportamientos de riesgo. En combinación con los análisis de regresión tradicionales, los análisis de redes podrían proporcionar información útil y poner de relieve factores fundamentales para disminuir el hábito de compartir agujas en personas que se inyectan droga.

Métodos: Se recolectaron datos sociodemográficos e información sobre las prácticas de inyección y prácticas sexuales en una encuesta transversal que se llevó a cabo en personas que se inyectaban droga en cinco prefecturas de la provincia de Yunnan, en China. Se utilizó una combinación de los análisis de regresión logística y de correlación de redes para explorar los factores claves para disminuir el hábito de compartir agujas en personas que se inyectan droga.

Resultados: En un total de 1049 personas que se inyectan droga, 37,5% tenían un historial de compartir agujas. El análisis logístico mostró que los residentes de Zhaotong, Qujing, Dehong, o Lincang, el uso de diazepam, una mayor duración de la inyección, la reutilización de agujas, y la infección por el virus del HIV, hepatitis viral, tuberculosis y/o malaria se asociaban de manera independiente al hábito de compartir agujas. Los análisis de correlación de redes mostraron que, en comparación con personas que se inyectan droga que nunca compartieron agujas, las personas que se inyectan droga y que comparten agujas lograrían la meta de disminuir el daño mas rápido y de manera más permanente. El estatus serológico de VIH y el estado civil estuvieron íntimamente asociados a otros factores de riesgo. La combinación del análisis de regresión con el análisis de redes, mostró que las personas que se inyectan droga y que son seropositivas para el VIH son un grupo objetivo ideal para los programas de reducción de daños.

Conclusión: El compartir agujas es común entre las personas que se inyectan droga en Yunnan, y los programas de reducción de daños podrían ayudar a las personas que se inyectan droga y que son seropositivas para el VIH a disminuir los comportamientos de riesgo y prevenir enfermedades que se transmiten por sangre.

Translated from English version into Spanish by Maria Alejandra Aguada, through

TRANSLATORS
WITHOUT BORDERS
